# All-cause mortality in the cohorts of the Spanish AIDS Research Network (RIS) compared with the general population: 1997–2010

**DOI:** 10.1186/1471-2334-13-382

**Published:** 2013-08-20

**Authors:** Victoria Hernando, Belen Alejos, Susana Monge, Juan Berenguer, Lourdes Anta, David Vinuesa, Rosario Palacios, Roberto Muga, Santiago Moreno, Inma Jarrin

**Affiliations:** 1 Red de Investigación en Sida, Centro Nacional de Epidemiología, Instituto de Salud Carlos III, Avda. Monforte de Lemos, Madrid 5 28029, Spain; 2 CIBER de Epidemiología y Salud Pública (CIBERESP), Madrid, Spain; 3 Hospital Universitario Gregorio Marañón, Madrid, Spain; 4 Hospital Universitario Carlos III, Madrid, Spain; 5 Hospital Universitario San Cecilio, Granada, Spain; 6 Hospital Universitario Virgen de la Victoria, Malaga, Spain; 7 Hospital Universitario Germans Trias I Pujol, Badalona, Spain; 8 Hospital Universitario Ramon y Cajal, Madrid, Spain

**Keywords:** Mortality rate, HIV infection, Standardized mortality ratios, Excess mortality

## Abstract

**Background:**

Combination antiretroviral therapy (cART) has produced significant changes in mortality of HIV-infected persons. Our objective was to estimate mortality rates, standardized mortality ratios and excess mortality rates of cohorts of the AIDS Research Network (RIS) (CoRIS-MD and CoRIS) compared to the general population.

**Methods:**

We analysed data of CoRIS-MD and CoRIS cohorts from 1997 to 2010. We calculated: (i) all-cause mortality rates, (ii) standardized mortality ratio (SMR) and (iii) excess mortality rates for both cohort for 100 person-years (py) of follow-up, comparing all-cause mortality with that of the general population of similar age and gender.

**Results:**

Between 1997 and 2010, 8,214 HIV positive subjects were included, 2,453 (29.9%) in CoRIS-MD and 5,761 (70.1%) in CoRIS and 294 deaths were registered. All-cause mortality rate was 1.02 (95% CI 0.91-1.15) per 100 py, SMR was 6.8 (95% CI 5.9-7.9) and excess mortality rate was 0.8 (95% CI 0.7-0.9) per 100 py. Mortality was higher in patients with AIDS, hepatitis C virus (HCV) co-infection, and those from CoRIS-MD cohort (1997–2003).

**Conclusion:**

Mortality among HIV-positive persons remains higher than that of the general population of similar age and sex, with significant differences depending on the history of AIDS or HCV coinfection.

## Background

Mortality of HIV-infected persons in Western countries has decreased significantly due to improvements in combined antiretroviral therapy (cART) [1,2]. Nevertheless it continues to be higher than in the general population [3-5], even in HIV-infected patients with good initial response to cART [6]. Global reduction in mortality has been achieved thanks to a decrease of AIDS-related deaths which has led to a greater relevance of other causes of death in relation to co-morbidities, such as hepatitis C virus (HCV) and/or hepatitis B virus (HBV) co-infections, drug abuse and cardiovascular diseases [2,7].

In Barcelona and Navarre, HIV-positive subjects were found to have a higher mortality compared to the general population [8,9] but no estimates are available for the whole country. Unlike other cohorts, in this work we have analyzed data of a cohort of persons with HIV infection recruited during a period where highly effective antiretroviral treatment is available and all patients are naïve to treatment. But we believe that even though these patients may be in a better starting point than patients in other similar studies, the risk of mortality compared with the general population is still higher.

Therefore, the objectives of this study were to calculate the overall mortality rates, standardized mortality ratios (SMR), and excess mortality rates in the cohorts of the Spanish AIDS Research Network (RIS) – CoRIS-MD and CoRIS, comparing the overall mortality rates observed in HIV positive subjects in both cohorts with the mortality rates of the general population of similar age and sex.

## Methods

### Patients

We analyzed data from the cohorts of HIV-infected adults of the Spanish AIDS Research Network (RIS). CoRIS-MD is a multicenter cohort including data from 1997 to 2003 from 9 hospitals of 7 Spanish Autonomous regions assembled in 2003. CoRIS is a multicenter cohort which recruits patients from 2004 onwards from 28 health-care centers and hospitals in 12 of the 17 Autonomous regions that compose Spain [10,11]. Both cohorts recruit patients newly attended in any of the participating sites. Ethics approval was obtained from all hospitals Ethics’ Committees (see Appendix 1 all hospitals participants) and every patient provides written informed consent to participate in the cohorts. For this analysis, we selected subjects who were naïve to cART at cohort entry, older than 20 years, had a follow up of more than 6 months and had had at least one diagnostic test for hepatitis C virus.

### Variables

We considered the following variables: age at cohort entry (20–29; 30–39; 40–49; > = 50); gender (male, female); year of cohort entry; HIV transmission category, classified as injecting drugs users (IDUs), men who have sex with men (MSM), heterosexual contact and others or unknown risk category; AIDS before entry and changes in AIDS status during follow-up; CD4 count at entry (<200, 200–349, ≥350); HIV viral load at entry (<20000, 20000–100000, ≥100000); combined antiretroviral treatment (cART) initiation during follow-up; HCV serological status classified as positive or negative antibodies and vital status.

To calculate mortality rates, AIDS variable was classified as “Yes” when the person had AIDS before entering the cohort, AIDS at cohort entry or AIDS during follow-up and “No” when the person didn’t develop AIDS at any moment during the study.

### Statistical analyses

Descriptive analysis of patients’ characteristics was carried out using frequency distribution for categorical variables and median (interquartile range -IQR) for continuous variables.

Individuals were followed up from study entry to death, last study contact or the administrative censoring date (31/12/2003 in CoRIS-MD and 31/12/2010 in CoRIS) whichever arose first. We calculated mortality rates, overall and according to socio-demographic and clinical characteristics, as the number of deaths by 100 persons-year (py) of follow-up with 95% confidence intervals (95% CI) calculated using the exact Poisson method.

Standardized mortality ratios (SMR) were estimated for all-cause mortality in CoRIS-MD and CoRIS, comparing with the overall mortality rates of the general population standardized by sex and age. SMR were estimated as the ratio of observed deaths to expected deaths, had our patients had the same distribution of mortality as the general population. SMR were calculated through Poisson models offsetting expected mortality rates, and adjusted for gender, age, category of transmission and HCV test. Mortality rates for general population, between 1997 and 2010, were obtained from the National Statistics Institute (http://www.ine.es), stratified by sex and age at 5 year intervals. A constant mortality rate within each 5 year stratum was assumed.

A sensitivity analysis was performed to assess a possible *selection bias*. The SMR was calculated for the first 12 months after cohort entry separately for all patients together. This was to determinate whether it is necessary to include a lag time to avoid an overestimation of SMR.

Excess Mortality Rates were calculated as the difference between observed and expected deaths according to mortality in the general population, divided by the number of persons-year (py) of follow-up. Confidence intervals for Excess Mortality Rates were estimated using Poisson’s exact method.

All statistical analyses were performed by using Stata software (Version 11.0, College Station, Texas).

## Results

### Baseline characteristics of the study population

A total of 8,214 subjects were included in the study, 2,453 (29.9%) in CoRIS-MD and 5,761 (70.1%) in CoRIS, adding up to 28,743 persons-year of follow up, and 294 deaths.

Men represented 78.0% (n = 6,412) of the sample, and median age at the cohort entry was 35.0 years (interquartile range IQR: 30.2 – 41.0), 35.5 years (IQR: 30.2-41.7) for men and 34.2 years (IQR: 29.1-40.1) for women. Regarding transmission categories, the sample was distributed between injecting drugs users (IDUs) or ex-users, 25.0% (n = 2,050), men who have sex with men (MSM), 39.6% (n = 3,255), and heterosexuals, 30.7% (n = 2,524). A 20.4% of the subjects had a history of an AIDS defining illness (ADI), although for 59.4% (n = 994) of them the ADI diagnosis was previous to cohort entry. Median CD4 count at cohort entry was 350 cell/mm^3^ (IQR 170 – 552), and median viral load was 39,811 copies/ml (IQR 7,520 – 135,988) (Table 1).

**Table 1:** Socio demographics and clinical characteristics at cohort entry for total of analyzed subjects and deceased subjects

		**Total**		**Deaths**	
	**py**	**n**	**%**	**n**	**%**
Total	28,743	8,214	100	294	100
Gender					
Males	21,903	6,412	78.0	237	80.6
Females	6,840	1,802	22.0	57	19.4
Age at cohort entry (years)					
20–29	6,945	2,064	25.1	34	11.6
30–39	13,778	3,722	45.3	145	49.3
40–49	5,584	1,705	13.1	71	24.1
> = 50	2,436	723	8.8	44	15.0
Median age (IQR)		35.0 (30.2–41.0)		37.7(33.5–44.5)	
Category of transmission					
IDUs	8,515	2,050	25.0	177	60.2
MSM	9,994	3,255	39.6	41	14.0
Heterosexual	8,909	2,524	30.7	67	22.8
Others/Unknown	1,325	385	4.7	9	3.0
AIDS					
No	22,255	6,542	79.6	144	49.0
AIDS before entry	3,667	994	12.1	72	24.5
AIDS after entry	2,821	678	8.3	78	26.5
CD4 count at entry (cel/mm^3^)					
<200	7,525	2,217	27.0	141	48.0
200–349	5,191	1,567	19.1	39	13.3
> = 350	12,366	3,744	45.6	64	21.8
Unknown	3,661	686	8.4	50	17.0
Median (IQR)		350 (170–552)		154 (66–390)	
HIV viral load (copies/ml)					
<20.000	8,748	2,769	33.7	61	20.7
20.000-100.000	7,141	2,202	26.8	65	22.1
>100.000	7,181	2,196	26.7	89	30.3
Unknown	5,673	1,047	12.8	79	26.9
Median (IQR)		39,810 (7,520–135,988)		78,200 (17,335–230,000)	
Cohorts					
CoRIS (2004–2008)	18,447	5,761	70.1	137	46.6
CoRIS-MD (1997–2003)	10,296	2,453	29.9	157	53.4
HCV test					
Negative	18,332	5,673	69.1	96	32.6
Positive	10,411	2,541	30.9	198	67.4
Antiretroviral treatment during follow-up					
No	9,992	1,948	23.7	63	21.4
Yes	18,751	6,266	76.3	231	78.6

*IDUs* Injecting Drugs Users, *MSM* Men have Sex with Men, *HCV* Hepatitis C virus.

Among the 294 deceased subjects, 80.6% (n = 237) were men, and median age was 37.7 years (IQR 33.3 – 44.5). Some 60.2% (n = 177) were IDU or ex-IDU, 51.0% (n = 150) had an AIDS diagnosis and 67.4% (n = 198) were co-infected by HCV. Median CD4 count at entry was 154 cell/mm^3^ (IQR 66 – 390) and median HIV viral load was 78,200 copies/ml (IQR 17,335 – 230,000) (Table 1).

### Mortality rates, standardized mortality ratios and excess mortality rates

**Figure 1:**
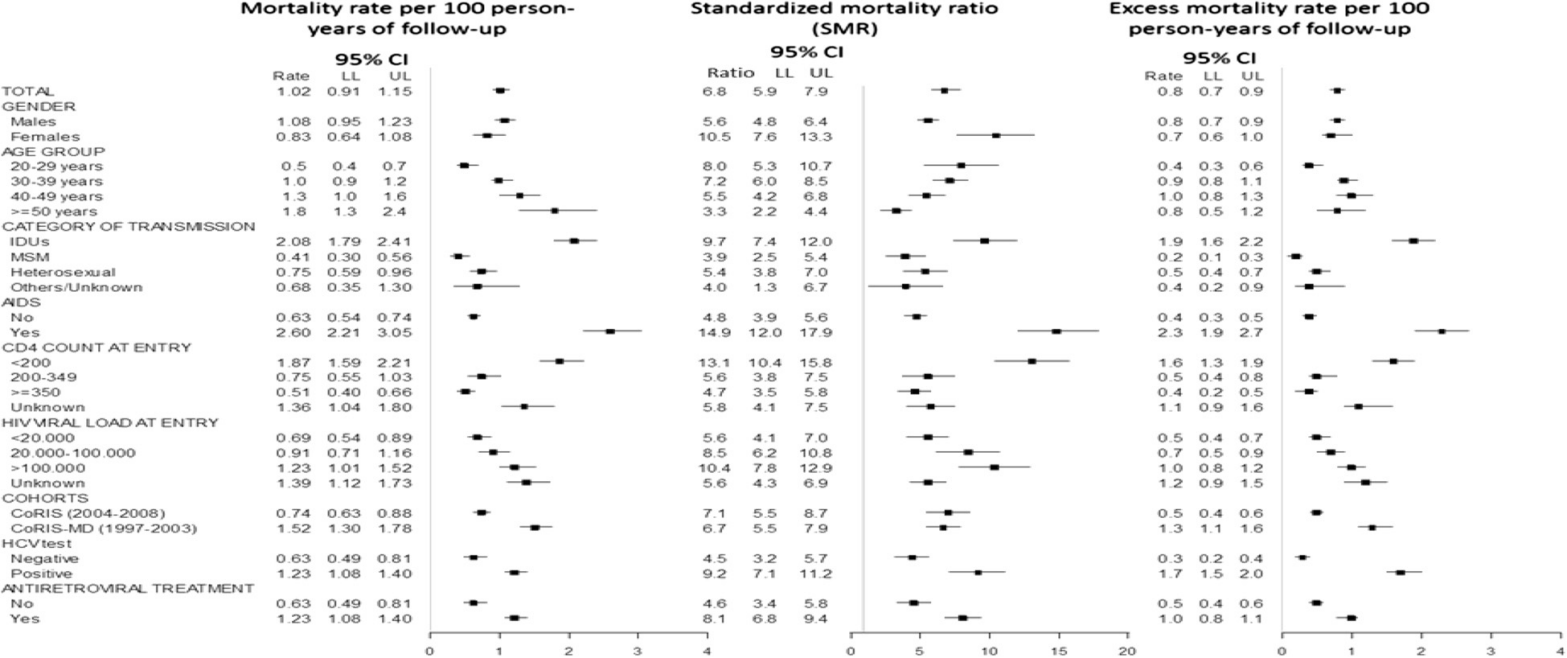
Mortality rates per 100 person-years of follow-up, Standardized mortality ratio (SMRs) and excess mortality rates per 100 person-years of follow-up according to sociodemographic, epidemiological and clinical characteristics.

Figure 1 shows mortality rates for 100 persons-year (py) of follow up, standardized mortality ratios and excess mortality rates for 100 py in both RIS cohorts.

Overall mortality rate was 1.02 (95% CI: 0.91-1.15) deaths for 100 py of follow up, higher for men (1.08; 95% CI: 0.95-1.23), for subjects over 50 years-old (1.81; 95% CI: 1.34-2.42), for IDU (2.08; 95% CI: 1.79-2.41) compared to both MSM (0.41; 95% CI: 0.30-0.56) and heterosexuals (0.75; 95% CI: 0.59-0.96) and for patients included in CoRIS-MD (1.52; 95% CI: 1.30-1.78). For patients who had an AIDS diagnosis, mortality rate was 2.06 (95% CI: 2.21-3.05), compared to 0.63 (95% CI: 0.54-0.74) for those who were AIDS-free. For HCV co-infected patients mortality rate rose up to 1.90 (95% CI: 1.65-2.19) in contrast with 0.52 (95% CI: 0.42-0.64) for those not co-infected.

Global mortality in both CoRIS cohorts was 6.8 (95% CI: 5.9-7.9) times higher than mortality of the general population of same age and sex. As opposed to the crude mortality rates, standardized mortality ratios were higher in women (10.5; 95% CI: 7.6-13.3) compared to men (5.6; 95% CI: 4.8-6.4). Still, a higher SMR was found for IDUs (9.7; 95% CI: 7.4-12.0), persons with an AIDS diagnosis (14.9; 95% CI: 12.0-17.9), persons co-infected with HCV (9.2; 95% CI: 7.1-11.2) and those receiving antiretroviral treatment (8.1; 95% CI: 6.8-9.4).

In the sensitivity analysis, considering only the first 12 month of follow-up, SMR is lower than in the complete analysis (4.0; 95% CI 2.4 -5.6).

Finally, regarding excess mortality rate, as an absolute estimator, results are similar to those observed for crude mortality rates (Figure 1).

## Discussion and conclusion

Our results show that all-cause mortality in CoRIS-MD and CoRIS cohorts, between 1997 and 2010, is close to seven times higher than that of the general population of the same age and sex. Significant differences have been found depending on the history of AIDS and HCV co-infection.

A previously published study, carried out in similar cohorts in Europe and North America, found a lower global SMR, of 3.36 (95% CI: 3.16 – 3.56), but with a notable heterogeneity between cohorts depending on participant-specific characteristics, and being higher for cohorts with a greater representation of IDUs [12]. For example, Aldaz et al. found mortality of HIV-infected persons in Navarre (Spain) to be 14 times higher than mortality in general population; 63% of this cohort had been infected through the use of injected drugs [8].

These differences could also be related to the higher prevalence of HCV-co-infection as the standardized mortality in HCV co-infected subjects in our study was 9.2 times higher than the general population’s. Similar results were found by Lewden et al., where SMR for HCV co-infected persons were 13.9 compared to 4.4 for the HCV negative subjects [4]. In a previous study of CoRIS-MD and CoRIS cohorts, an important increase of the risk of both all cause mortality and liver-related mortality was observed for HIV patients coinfected with HCV [13]. Berenguer et al. also found a decrease in overall mortality in HIV patients in cART era, but only in HCV negative subjects [14] and Chen et al. in a meta-analysis found that the risk of mortality was increased in HCV/HIV coinfected patients in HAART era [15].

In our study, we found a similar SMR for patients recruited in CoRIS, from 2004 onwards, and those recruited in CoRIS-MD, from 1997 to 2003, after adjustment for gender, age, transmission category and HCV infection. That is, the difference in the subject’s characteristics along these years, the decrease in the representation of IDUs and the percentage of HCV co-infected subjects [11,16,17] were corrected after adjustment. Others studies observed a lower mortality in recent years with the improvement in antiretroviral therapies [18-21], although when specific groups were analyzed, for example: IDUs, found that mortality risk remain elevated [21].

We found non-statistically significant, lower mortality rates in women compared to men. Eventhough the women in our study showed a mortality ratio 10.5 times higher than women of the same age from the general population, and almost doubled the one from men in the cohorts. This higher relative mortality in women could be explained by the fact that women in the general population have a higher life expectancy than men, and specifically, mortality in the general population is very low in women between ages 30 to 40, where we find the majority of HIV-infected women [22]. The lower excess mortality rate in women is consistent with the higher proportion of HIV-infected men in the Spanish epidemic, and in our cohorts [23].

A possible limitation in the calculation of SMR could be using mortality rates in the general population to calculate the expected deaths, because this population contains HIV-related deaths. In our analysis, HIV-related mortality represents a small proportion of all-cause mortality in the general population of Spain, so therefore we consider correct to use the general population mortality rates to calculate the mortality rates in a non-HIV infected population.

The sensitivity analysis shows that when we establish as inclusion criteria to have at least 6 months of follow-up, we are introducing a time window to avoid the selection bias indirectly and overestimate SMRs.

To conclude, mortality in HIV-infected persons continues to be higher than that of the general population, although it has decreased in recent years. For future studies, we would highly recommend to consider, along with global mortality, excess mortality rate for specific causes of death, such as hepatic, non-aids related malignancies or drug-related, especially among IDUs.

## Appendix 1: Centers and investigators involved in CoRIS

**Executive committee:** Juan Berenguer, Julia del Amo, Federico García, Félix Gutiérrez, Pablo Labarga, Santiago Moreno y María Ángeles Muñoz.

**Fieldwork, data management and analysis**: Paz Sobrino Vegas, Victoria Hernando Sebastián, Belén Alejos Ferreras, Débora Álvarez del Arco, Susana Monge Corella, Inma Jarrín Vera, Adela Castelló.

**BioBank:** M Ángeles Muñoz-Fernández, Isabel García-Merino, Coral Gómez Rico, Jorge Gallego de la Fuente and Almudena García Torre.

Participating centres:

Hospital General Universitario de Alicante (Alicante): Joaquín Portilla Sogorb, Esperanza Merino de Lucas, Sergio Reus Bañuls, Vicente Boix Martínez, Livia Giner Oncina, Carmen Gadea Pastor, Irene Portilla Tamarit, Patricia Arcaina Toledo.

Hospital Universitario de Canarias (Santa Cruz de Tenerife): Juan Luis Gómez Sirvent, Patricia Rodríguez Fortúnez, María Remedios Alemán Valls, María del Mar Alonso Socas, Ana María López Lirola, María Inmaculada Hernández Hernández, Felicitas Díaz-Flores.

Hospital Carlos III (Madrid): Vicente Soriano, Pablo Labarga, Pablo Barreiro, Pablo Rivas, Francisco Blanco, Luz Martín Carbonero, Eugenia Vispo, Carmen Solera.

Hospital Universitario Central de Asturias (Oviedo): Victor Asensi, Eulalia Valle, José Antonio Cartón

Hospital Clinic (Barcelona): José M. Miró, María López-Dieguez, Christian Manzardo, Laura Zamora, Iñaki Pérez, Mª Teresa García, Carmen Ligero, José Luis Blanco, Felipe García-Alcaide, Esteban Martínez, Josep Mallolas, José M. Gatell.

Hospital Doce de Octubre (Madrid): Rafael Rubio, Federico Pulido, Silvana Fiorante, Jara Llenas, Violeta Rodríguez, Mariano Matarranz.

Hospital Donostia (San Sebastián): José Antonio Iribarren, Julio Arrizabalaga, María José Aramburu, Xabier Camino, Francisco Rodríguez-Arrondo, Miguel Ángel von Wichmann, Lidia Pascual Tomé, Miguel Ángel Goenaga, Mª Jesús Bustinduy, Harkaitz Azkune Galparsoro.

Hospital General Universitario de Elche (Elche): Félix Gutiérrez, Mar Masiá, Cristina López Rodríguez, Sergio Padilla, Andrés Navarro, Fernando Montolio, Catalina Robledano García, Joan Gregori Colomé.

Hospital Germans Trías i Pujol (Badalona): Bonaventura Clotet, Cristina Tural, Lidia Ruiz, Cristina Miranda, Roberto Muga, Jordi Tor, Arantza Sanvisens.

Hospital General Universitario Gregorio Marañón (Madrid): Juan Berenguer, Juan Carlos López Bernaldo de Quirós, Pilar Miralles, Jaime Cosín Ochaíta, Isabel Gutiérrez Cuellar, Margarita Ramírez Schacke, Belén Padilla Ortega, Paloma Gijón Vidaurreta, Ana Carrero Gras, Teresa Aldamiz-Echevarría Lois y Francisco Tejerina Picado.

Hospital Universitari de Tarragona Joan XXIII, IISPV, Universitat Rovira i Virgili (Tarragona): Francesc Vidal, Joaquín Peraire, Consuelo Viladés, Sergio Veloso, Montserrat Vargas, Miguel López-Dupla, Montserrat Olona, Alba Aguilar, Joan Josep Sirvent, Verónica Alba, Olga Calavia.

Hospital Universitario La Fe (Valencia): José López Aldeguer, Marino Blanes Juliá, José Lacruz Rodrigo, Miguel Salavert, Marta Montero, Eva Calabuig, Sandra Cuéllar.

Hospital Universitario La Paz (Madrid): Juan González García, Ignacio Bernardino de la Serna, José Ramón Arribas López, María Luisa Montes Ramírez, Jose Mª Peña, Blanca Arribas, Juan Miguel Castro, Fco Javier Zamora Vargas, Ignacio Pérez Valero, Miriam Estébanez, Silvia García Bujalance, Marta Díaz.

Hospital de la Princesa (Madrid): Ignacio de los Santos, Jesús Sanz Sanz, Ana Salas Aparicio, Cristina Sarriá Cepeda.

Hospital San Pedro-CIBIR (Logroño): José Antonio Oteo, José Ramón Blanco, Valvanera Ibarra, Luis Metola, Mercedes Sanz, Laura Pérez-Martínez.

Hospital San Pedro II (Logroño): Javier Pinilla Moraza.

Hospital Universitario Mutua de Terrassa (Terrassa): David Dalmau, Angels Jaén Manzanera, Mireia Cairó Llobell, Daniel Irigoyen Puig, Laura Ibáñez, Queralt Jordano Montañez, Mariona Xercavins Valls, Javier Martinez-Lacasa, Pablo Velli, Roser Font.

Hospital de Navarra (Pamplona): María Rivero, Marina Itziar Casado, Jorge Alberto Díaz González, Javier Uriz, Jesús Repáraz, Carmen Irigoyen, María Jesús Arraiza.

Hospital Parc Taulí (Sabadell): Ferrán Segura, María José Amengual, Eva Penelo, Gemma Navarro, Montserrat Sala, Manuel Cervantes, Valentín Pineda.

Hospital Ramón y Cajal (Madrid): Santiago Moreno, José Luis Casado, Fernando Dronda, Ana Moreno, María Jesús Pérez Elías, Dolores López, Carolina Gutiérrez, Beatriz Hernández, María Pumares, Paloma Martí.

Hospital Reina Sofía (Murcia): Alfredo Cano Sánchez, Enrique Bernal Morell, Ángeles Muñoz Pérez.

Hospital San Cecilio (Granada): Federico García García, José Hernández Quero, Alejandro Peña Monje, Leopoldo Muñoz Medina, Jorge Parra Ruiz.

Centro Sanitario Sandoval (Madrid): Jorge Del Romero Guerrero, Carmen Rodríguez Martín, Teresa Puerta López, Juan Carlos Carrió Montiel, Cristina González, Mar Vera.

Hospital Universitario Santiago de Compostela (Santiago de Compostela): Antonio Antela, Arturo Prieto, Elena Losada.

Hospital Son Espases (Palma de Mallorca): Melchor Riera, Javier Murillas, Maria Peñaranda, Maria Leyes, Mª Angels Ribas, Antoni Campins, Concepcion Villalonga, Carmen Vidal.

Hospital Universitario de Valme (Sevilla): Juan Antonio Pineda, Eva Recio Sánchez, Fernando Lozano de León, Juan Macías, José del Valle, Jesús Gómez-Mateos.

Hospital Virgen de la Victoria (Málaga): Jesús Santos González, Manuel Márquez Solero, Isabel Viciana Ramos, Rosario Palacios Muñoz.

Hospital Universitario Virgen del Rocío (Sevilla): Pompeyo Viciana, Manuel Leal, Luis Fernando López-Cortés, Mónica Trastoy.

## Competing interests

The authors declare that they have no competing interests.

## Authors’ contributions

VH, BA, SM and IJ were involved in designing the study, participated in the collection and analysis of the data. VH, BA and IJ wrote the first draft if the manuscript. All authors contributed to data collection, reviewed draft of the manuscript and approved the final manuscript.

## Pre-publication history

The pre-publication history for this paper can be accessed here:

http://www.biomedcentral.com/1471-2334/13/382/prepub

## References

[B1] Krentz HB Kliewer G Gill MJ Changing mortality rates and causes of death for HIV-infected individuals living in Southern Alberta, Canada from 1984 to 2003 HIV Med 2005 6 99 106 10.1111/j.1468-1293.2005.00271.x 15807715

[B2] Palella FJ Jr Baker RK Moorman AC Chmiel JS Wood KC Brooks JT Holmberg SD Mortality in the highly active antiretroviral therapy era: changing causes of death and disease in the HIV outpatient study J Acquir Immune Defic Syndr 2006 43 27 34 10.1097/01.qai.0000233310.90484.16 16878047

[B3] Jaggy C Von Overbeck J Ledergerber B Schwarz C Egger M Rickenbach M Furrer HJ Telenti A Battegay M Flepp M Mortality in the Swiss HIV Cohort Study (SHCS) and the Swiss general population Lancet 2003 362 877 878 10.1016/S0140-6736(03)14307-3 13678976

[B4] Lewden C Chene G Morlat P Raffi F Dupon M Dellamonica P Pellegrin JL Katlama C Dabis F Leport C HIV-infected adults with a CD4 cell count greater than 500 cells/mm3 on long-term combination antiretroviral therapy reach same mortality rates as the general population J Acquir Immune Defic Syndr 2007 46 72 77 10.1097/QAI.0b013e3181576818 17621240

[B5] Lohse N Hansen AB Pedersen G Kronborg G Gerstoft J Sorensen HT Vaeth M Obel N Survival of persons with and without HIV infection in Denmark, 1995–2005 Ann Intern Med 2007 146 87 95 10.7326/0003-4819-146-2-200701160-00003 17227932

[B6] Van Sighem A Danner S Ghani AC Gras L Anderson RM De Wolf F Mortality in patients with successful initial response to highly active antiretroviral therapy is still higher than in non-HIV-infected individuals J Acquir Immune Defic Syndr 2005 40 212 218 10.1097/01.qai.0000165911.97085.d0 16186740

[B7] Causes of death in HIV-1-infected patients treated with antiretroviral therapy, 1996–2006: collaborative analysis of 13 HIV cohort studies Clin Infect Dis 2010 50 1387 1396 20380565 10.1086/652283 PMC3157754

[B8] Aldaz P Moreno-Iribas C Egues N Irisarri F Floristan Y Sola-Boneta J Martinez-Artola V Sagredo M Castilla J Mortality by causes in HIV-infected adults: comparison with the general population BMC Public Health 2011 11 300 10.1186/1471-2458-11-300 21569323 PMC3112125

[B9] Martinez E Milinkovic A Buira E De Lazzari E Leon A Larrousse M Lonca M Laguno M Blanco JL Mallolas J Incidence and causes of death in HIV-infected persons receiving highly active antiretroviral therapy compared with estimates for the general population of similar age and from the same geographical area HIV Med 2007 8 251 258 10.1111/j.1468-1293.2007.00468.x 17461853

[B10] Caro-Murillo AM Castilla J Perez-Hoyos S Miro JM Podzamczer D Rubio R Riera M Viciana P Lopez AJ Iribarren JA Spanish cohort of naive HIV-infected patients (CoRIS): rationale, organization and initial results Enferm Infecc Microbiol Clin 2007 25 23 31 10.1157/13096749 17261243

[B11] Sobrino-Vegas P Gutierrez F Berenguer J Labarga P Garcia F Alejos-Ferreras B Munoz MA Moreno S Del Amo J [The cohort of the spanish hiv research network (coris) and its associated biobank; organizational issues, main findings and losses to follow-up] Enferm Infecc Microbiol Clin 2011 29 645 653 10.1016/j.eimc.2011.06.002 21820763

[B12] Zwahlen M Harris R May M Hogg R Costagliola D De Wolf F Gill J Fatkenheuer G Lewden C Saag M Mortality of HIV-infected patients starting potent antiretroviral therapy: comparison with the general population in nine industrialized countries Int J Epidemiol 2009 38 1624 1633 19820106 10.1093/ije/dyp306 PMC3119390

[B13] Hernando V Perez-Cachafeiro S Lewden C Gonzalez J Segura F Oteo JA Rubio R Dalmau D Moreno S Amo JD All-cause and liver-related mortality in HIV positive subjects compared to the general population: differences by HCV co-infection J Hepatol 2012 57 743 751 10.1016/j.jhep.2012.06.010 22709620

[B14] Berenguer J Alejos B Hernando V Viciana P Salavert M Santos I Gomez-Sirvent JL Vidal F Portilla J Del AJ Trends in mortality according to hepatitis C virus serostatus in the era of combination antiretroviral therapy AIDS 2012 26 17 2241 2246 10.1097/QAD.0b013e3283574e94 22781223

[B15] Chen TY Ding EL Seage Iii GR Kim AY Meta-analysis: increased mortality associated with hepatitis C in HIV-infected persons is unrelated to HIV disease progression Clin Infect Dis 2009 49 1605 1615 10.1086/644771 19842982 PMC2805261

[B16] Gutierrez F Padilla S Masia M Iribarren JA Moreno S Viciana P Munoz L Gomez Sirvent JL Vidal F Lopez-Aldeguer J Clinical outcome of HIV-infected patients with sustained virologic response to antiretroviral therapy: long-term follow-up of a multicenter cohort PLoS One 2006 1 e89 10.1371/journal.pone.0000089 17183720 PMC1762396

[B17] Perez CS Del Amo J Iribarren JA Salavert LM Gutierrez F Moreno A Labarga P Pineda JA Vidal F Berenguer J Decrease in serial prevalence of coinfection with hepatitis C virus among HIV-infected patients in Spain, 1997–2006 Clin Infect Dis 2009 48 1467 1470 10.1086/598333 19368502

[B18] Ray M Logan R Sterne JA Hernandez-Diaz S Robins JM Sabin C Bansi L Van Sighem A De Wolf F Costagliola D The effect of combined antiretroviral therapy on the overall mortality of HIV-infected individuals AIDS 2010 24 123 137 19770621 10.1097/QAD.0b013e3283324283 PMC2920287

[B19] Smit C Geskus R Walker S Sabin C Coutinho R Porter K Prins M Effective therapy has altered the spectrum of cause-specific mortality following HIV seroconversion AIDS 2006 20 741 749 10.1097/01.aids.0000216375.99560.a2 16514305

[B20] Sobrino-Vegas P Garcia-San Miguel L Caro-Murillo AM Miro JM Viciana P Tural C Saumoy M Santos I Sola J Del Amo J Delayed diagnosis of HIV infection in a multicenter cohort: prevalence, risk factors, response to HAART and impact on mortality Curr HIV Res 2009 7 224 230 10.2174/157016209787581535 19275591

[B21] Lewden C Bouteloup V De WS Sabin C Mocroft A Wasmuth JC Van SA Kirk O Obel N Panos G All-cause mortality in treated HIV-infected adults with CD4 >/=500/mm3 compared with the general population: evidence from a large European observational cohort collaboration Int J Epidemiol 2012 41 433 445 22493325 10.1093/ije/dyr164

[B22] Ministry of Health SPaE Patterns of mortality in Spain, 2008 2011 http://www.mspsi.gob.es/estadEstudios/estadisticas/estadisticas/estMinisterio/mortalidad/mortalidad.htm

[B23] Centro Nacional de Epidemiología Vigilancia Epidemiológica del VIH/SIDA en España 2011 http://www.isciii.es/ISCIII/es/contenidos/fd-servicios-cientifico-tecnicos/fd-vigilancias-alertas/fd-enfermedades/Informe_VIH-sida_Junio_2011.pdf23515771

